# Cross-sectional and longitudinal association between accelerometer-measured light-intensity physical activity and cognitive function in older adults

**DOI:** 10.3389/fnagi.2025.1505172

**Published:** 2025-03-13

**Authors:** Jing-Han Jhan, Jiaren Chen, Ting-Fu Lai, Jong-Hwan Park, Yung Liao

**Affiliations:** ^1^Graduate Institute of Sport, Leisure and Hospitality Management, National Taiwan Normal University, Taipei City, Taiwan; ^2^Biomedical Research Institute, Pusan National University Hospital, Busan, Republic of Korea; ^3^Department of Convergence Medicine, Pusan National University School of Medicine, Yangsan, Republic of Korea; ^4^Department of Clinical Bio-Convergence, Graduate School of Convergence in Biomedical Science, Pusan National University School of Medicine, Yangsan, Republic of Korea; ^5^Convergence Medical Institute of Technology, Pusan National University Hospital, Busan, Republic of Korea; ^6^Faculty of Sport Sciences, Waseda University, Tokorozawa, Japan

**Keywords:** light-intensity physical activity, older adults, cognitive function, accelerometer, MMSE

## Abstract

**Objectives:**

Regarding the methods of improving cognitive function in older adults, it is well-established that moderate-to-vigorous physical activity (MVPA) is beneficial. Considering the safety and mobility of older adults, recent research has focused on the benefits of light-intensity physical activity (LPA) on cognitive function. However, limited research has utilized the different domains of cognitive examination scales [such as the Mini-Mental State Examination (MMSE)] to analyze the relationship between LPA and different domains of cognitive function and compare the cross-sectional and longitudinal results. Thus, this study aimed to investigate the cross-sectional and longitudinal association between LPA and both overall and domain-specific cognitive function in older Taiwanese adults.

**Methods:**

This longitudinal study recruited participants in an outpatient department of geriatrics and gerontology in a medical center in Taipei City, Taiwan. Data was collected from September 2020 to 2021; the follow-up data were collected until December 2022. Participants were community-dwelling older adults aged ≥ 65 years who could walk independently. Baseline physical activity (any bodily movement produced by skeletal muscles that requires energy expenditure) and sedentary behavior (any waking behavior while in a sitting, reclining or lying posture with low energy expenditure) were measured with a GT3X+ triaxial accelerometer, categorized as sedentary behavior (< 100 counts/min), LPA (100–2,019 counts/min) and MVPA (≥ 2,020 counts/min). Cognitive functions were measured using the Chinese version of MMSE for the baseline and follow-up data. Binary logistic regression analysis was used to examine the association between 3 h/day of LPA and cognitive functions. Baseline dependent variables were whether participants had overall cognitive impairment and whether scores of domain-specific MMSE were at the maximum level; in the follow-up analysis, the dependent variables were whether overall and domain-specific scores of MMSE maintained or increased (obtained by subtracting the baseline from the follow-up overall and individual domain MMSE scores).

**Results:**

A total of 167 participants were included (52.10% female; 76.11 ± 6.47 years). The cross-sectional analysis results indicated that in the adjusted model (adjusted for age, sex, educational degree, wear time, MVPA time, and sedentary behavior time), both overall and domain-specific cognitive functions were not significantly associated with ≥ 3 h/day of LPA. The longitudinal analysis results indicated that in the adjusted model, ≥ 3 h/day of LPA was significantly negatively associated with the maintenance or increase of language [odds ratio (OR): 0.88; 95% confidence interval (CI): 0.01–0.99; *P* = 0.049], and significantly positively associated with the maintenance or increase of orientation (OR: 3.83; 95% CI: 1.01–14.46; *P* = 0.048).

**Conclusion:**

The cross-sectional and longitudinal impacts of engaging in ≥ 3 h/day of LPA on cognitive functions differed. While engaging in ≥ 3 h/day of LPA has no significant short-term benefits, performing ≥ 3 h/day of LPA is beneficial for maintaining or improving orientation cognitive function in long term. Further studies should explore the longitudinal relationship between LPA and orientation cognitive function to provide a more comprehensive understanding of their potential interactions.

## 1 Introduction

Lead by the decline in fertility and increase in longevity, population aging has become a critical issue worldwide. It is anticipated that Taiwan will enter the status of a super-aged society by 2050 (National Development Council, 2022). Aging-related diseases include neurodegenerative, cardiovascular, and metabolic diseases; Alzheimer’s disease (AD), the most prevalent form of dementia, is a progressive neurological disorder that commonly occurs in older adults (Guo et al., 2022). A major symptom of dementia is the loss of cognitive function, causing problems with language skills, visual perception, or paying attention in daily life (National Institutes of Health, 2020). In Taiwan, the prevalence rate of dementia among older adults aged ≥ 65 years is 7.99%, and it is projected that the number of people aged ≥ 65 years with dementia will become nearly 680,000 in < 20 years (Ministry of Health and Welfare, 2024). Based on previous research, dementia — especially among those with chronic diseases — inconveniences the daily lives of those affected and incurs large medical expenses and burdens on caregivers (Chang, 2016; Ku et al., 2016). Dementia is the last and the most severe stage of cognitive impairment (Alzheimer’s Association, 2022). Specifically, the progression of cognitive impairment is categorized as normal cognition, prodromal dementia, and dementia (Golomb et al., 2004). While some individuals are diagnosed with Mild Cognitive Impairment (MCI), the majority tend to decline, with most of these declining patients eventually being diagnosed with AD (Golomb et al., 2004). Hence, the severity of cognitive impairment needs to be emphasized. To impede the trend of cognitive function deterioration, concerns regarding the cognitive function of older adults in Taiwan should be seriously considered.

Risk factors for cognitive function impairment can be classified as non-modifiable (including age, sex, and family medical history) or modifiable [including educational attainment, physical activity (PA), tobacco use, certain medical conditions, and social isolation] (World Health Organization, 2019). Among these risk factors, modifiable risk factors are targeted to delay the progression of cognitive impairment (World Health Organization, 2019). According to some cross-sectional and systematic reviews, physical activity (PA) is a crucial factor that has a positive impact on cognitive functions among older adults and can be incorporated into daily life (Coll-Padrós et al., 2019; de Frutos-Lucas et al., 2020; Feng et al., 2019; Kennedy et al., 2017; Kim et al., 2022; Livingston et al., 2020; Mc Ardle et al., 2023; Mellow et al., 2022; Pengpid and Peltzer, 2022; Veronese et al., 2023). Physical activity was defined as any bodily movement produced by skeletal muscles that requires energy expenditure (World Health Organization, 2024), categorized into different intensities referring to the rate of metabolic energy demand during exercise (MacIntosh et al., 2021). Measurements of physical activity can be divided into objective measurements and subjective measurements. Compared to subjective ones, objective measurements can avoid overestimating physical activity (Lee et al., 2011). It was previously reported that engaging in moderate-to-vigorous intensity PA (MVPA) had positive effects on cognitive function among older adults (Livingston et al., 2020); more specifically, it was demonstrated that engaging in 150 min of MVPA per week can enhance cognitive function among older adults (O’Brien et al., 2021). However, considering the difficulty of MVPA and the safety of older adults, light-intensity PA (LPA) — including casual walking, lifting lightweight objects, light household chores or yard works, and stretching — is more realistically achievable and feasible to accomplish among older adults (Tse et al., 2015).

Although most relevant research has concentrated on the association between MVPA or total PA and cognitive function (Mellow et al., 2022; Sofi et al., 2011; Zhu et al., 2017), increasing research have proven that LPA is beneficial for cognitive function in older adults (Amagasa et al., 2018; Rojer et al., 2021). Additionally, previous research showed that LPA and overall cognitive function were positively related regardless of whether LPA was assessed by objective instruments or questionnaires (Hsiao et al., 2022; Lee et al., 2013; Stubbs et al., 2017; Wu et al., 2020), while cognitive function examination scales including the Mini-Mental State Examination (MMSE), Montreal Cognitive Assessment (MoCA) and Ascertain Dementia 8-item Questionnaire (AD8) were used. Studies further proposed specific time threshold for engaging in LPA—engaging in ≥ 3 h/day LPA is associated with reduced risks of all-cause mortality (Ku et al., 2020) and engaging in ≥ 3 h of LPA is beneficial for cognitive function (Hsiao et al., 2022), providing clear time indicators for engaging in LPA. As for the domain-specific measurements of cognitive function, a scoping review reported that cognitive function testing could be categorized into memory (Verbal Digit Span-Forward test), attention and processing speed (Symbol Search task, Symbol Coding task and Trail Making test), executive function (Task-switching paradigm), and overall cognitive function (Telephonic assessment and interview) (Erlenbach et al., 2020). Adding to that, LPA was reported to be positively related to memory, attention, and executive function (Erlenbach et al., 2020).

In summary, most previous studies that used objective instruments and various cognitive examination scales and tests indicated a positive relationship between LPA and both overall and domain-specific cognitive functions (Amagasa et al., 2018; Erlenbach et al., 2020; Hsiao et al., 2022; Lee et al., 2013; Stubbs et al., 2017). However, limited research utilized the different domains of cognitive examination scales, such as MMSE, and accordingly focused on domain-specific cognitive functions to analyze the relationship between LPA and different domains of cognitive function. Furthermore, seldom has research explained the inconsistencies between cross-sectional and longitudinal results, analyzing the different effects of LPA on cognitive function in the short- and long-term.

This study aimed to investigate the cross-sectional and longitudinal associations between LPA and overall and different domains of cognitive function among community-dwelling older adults in Taiwan, and to compare the results of these two associations. Given that LPA is a possible protective factor for overall cognitive function, we hypothesized that engagement in 3 h/day of LPA was positively associated with older adults’ overall and domain-specific cognitive functions (measured using MMSE) in the short-term and long-term.

## 2 Materials and methods

### 2.1 Participants and study design

This study collected data from community-dwelling older adults aged ≥ 65 years who were able to walk independently (individuals with assistive devices walking and use of wheelchair were excluded). Participants were recruited from an outpatient department of geriatrics and gerontology in a medical center in Taipei City, Taiwan. Using convenience sampling, the interval between baseline and follow-up was at least 1 year—the baseline data were collected between September 2020 and September 2021; the follow-up data were collected until December 2022. All participants provided written informed consent and were informed of the detailed study process and purpose after doctors from the outpatient department assessed whether the participants met the recruitment criteria and were willing to participate in the study. Baseline data collection included: (i) a self-reported questionnaire (sociodemographic variables, health status, lifestyles behaviors and depressive symptom); (ii) accelerometer-assessed PA [wearing a triaxial accelerometer (GT3X+; ActiGraph, Pensacola, FL, United States) on either left or right side of waists based on personal preference for seven consecutive days] (Aadland and Ylvisåker, 2015); and (iii) cognitive function (MMSE test). The cognitive function test was conducted again during the follow-up survey.

**FIGURE 1:**
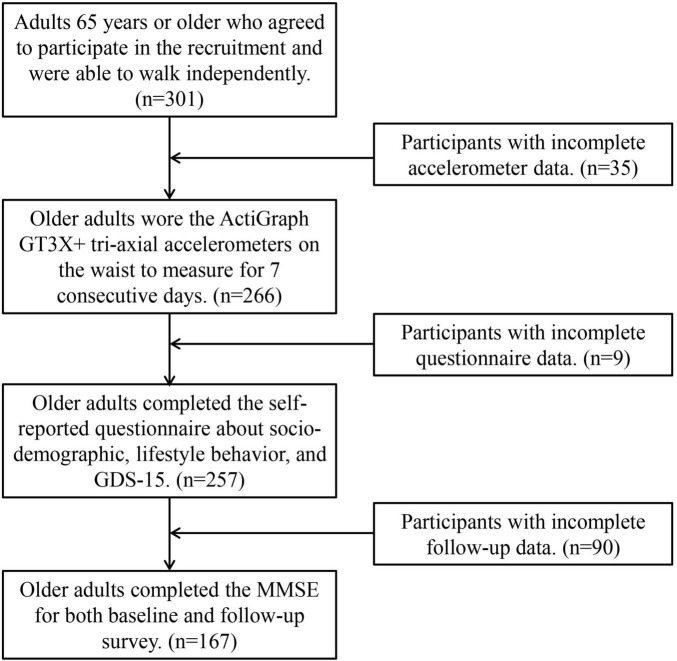
Flow chart of the research. GDS-15, 15-item geriatric depression scale; MMSE, Mini-Mental State Examination.

We conducted *a priori* analysis to estimate sample size via G*Power version 3.1.9.7 (Prajapati et al., 2010). The findings indicated that to achieve a statistical power of 0.80 in a binary logistic regression analysis examining the relationship between LPA and cognitive function, a minimum of 119 participants is required, assuming a significance level (α) of 0.05 (Prajapati et al., 2010). Initially, 301 participants were recruited. Participants with the following criteria were excluded: (1) did not meet the minimum criteria [wear ≥ 10 h per day, and at least four valid wear days (three weekdays and one weekend day)] for wearing the triaxial accelerometer (*n* = 35); (2) incompletely answered the self-reported questionnaires and Geriatric Depression Scale (GDS)-15 (*n* = 9); and (3) did not complete MMSE for both the baseline and follow-up survey (*n* = 90). After the data were screened by the exclusion criteria, 167 participants were enrolled in the final analysis. The recruitment procedure is shown in Figure 1. At the end of the study, participants were provided with an NTD 200 gift voucher, an incentive for study participation. This study was approved by the Research Ethics Committee of the National Taiwan University Hospital (202008046RINC).

### 2.2 Measures

#### 2.2.1 Cognitive function

For the baseline and follow-up data, this study measured the cognitive function of participants using the Chinese version of MMSE, a 30-point questionnaire containing 11 questions that are used to assess six domains of cognitive impairment, including orientation (10 points), attention and calculation (eight points), delayed recall (three points), language (five points), executive function (three points), and visuoconstructional skills (one points) (Folstein et al., 1975; Jia et al., 2021; Lin et al., 2019). In the baseline analysis, the dependent variables were whether participants had overall cognitive impairment (adjusted for education level) and whether scores of domain-specific MMSE were at the maximum level; in the follow-up analysis, the dependent variables were whether scores of MMSE were maintained or increased (obtained by subtracting the baseline from the follow-up overall and individual domain MMSE scores). The Chinese version of MMSE has a good sensitivity and specificity of 0.84 and 0.86, respectively (Tsai et al., 2016). Participants with higher total scores had more normal cognitive functions. The cognitive impairment of individuals with no formal schooling was defined with MMSE scores of ≤ 16; that of individuals with elementary school qualifications was defined with scores of ≤ 20; and that of individuals with junior high school qualifications or higher was defined with scores of ≤ 23 (Guo et al., 1988).

#### 2.2.2 Physical activity levels and sedentary behavior

For the baseline data, participants were asked to wear a waist-worn triaxial accelerometer (GT3X+) to measure the times participants spent in sedentary behavior (< 100 counts/min), LPA (100–2019 counts/min) and MVPA (≥ 2020 counts/min) (Troiano et al., 2008). The accelerometer had to be worn for seven consecutive days, and removed during water-related activities, such as bathing or similar activities; in this case, participants were requested to record the non-wear time. The ActiGraph GT3X+ triaxial accelerometer was verified as having a high validity (Dobell et al., 2019; Hänggi et al., 2013; Kelly et al., 2013). Based on previous studies (Migueles et al., 2017), valid data for this study met the following criteria: (1) accelerometer was worn for at least four valid days, including three weekdays and one weekend day; (2) a valid day represents a day during which the participants wore the triaxial accelerometer for ≥ 600 min (10 h) in a single day. Non-wear time was defined as a zero count on the accelerometer for a continuous period ≥ 60 min. The cumulative time unit for triaxial accelerometer measurements (count) is called Epoch length. According to previous research (Migueles et al., 2017), older adults often use 60 s as the unit for cumulative time. Studies have demonstrated the benefits of 3 h/day of LPA for older adults (Hsiao et al., 2022; Ku et al., 2020). Therefore, this study adopted the 3 h/day cut-off points of LPA to examine whether there was the benefit to both overall and domain-specific cognitive functions. Valid data from accelerometers were transferred into ActiLife software (version 6.0; ActiGraph) for analysis.

### 2.3 Covariates

Self-reported questionnaires were used to assess potential covariates in this study, including sociodemographic characteristics, lifestyle behaviors, and depressive symptoms. Sociodemographic characteristics included sex (female or male), age, education level (no formal schooling, elementary school, and junior high school), living status (living alone or living with others), body mass index (BMI; the objectively measured weight in kilograms divided by the square of the objectively measured height in meters), and the number of chronic diseases (< 4 or ≥ 4). We classified BMI into four groups such as underweight (< 18.5 kg/*m*^2^), normal (18.5–23.9 kg/*m*^2^), overweight (24–26.9 kg/*m*^2^), and obesity (≥ 27 kg/*m*^2^) (Ministry of Health and Welfare, 2018). Lifestyle behaviors included smoking (yes or no) and use of alcohol (yes or no). According to a previous study (Rojer et al., 2021), independence from MPVA and sedentary behavior could ensure the benefits of LPA for cognitive function. Hence, objective accelerometer-measured MVPA and sedentary behavior were also considered as covariates. In line with the recommendations of the World Health Organization, older adults are recommended to engage in MVPA for ≥ 150 min/week. In this study, MVPA was divided into two groups: ≥ 150 min/week and < 150 min/week. Moreover, sedentary behavior was divided into two groups based on a previous study (Ku et al., 2019): ≤ 9 h/day and > 9 h/day. Depressive symptoms are also associated with cognitive impairment (Camacho-Conde and Galán-López, 2020); to assess depressive symptoms, we used the 15-item GDS. In accordance with previous research (Greenberg, 2012), this covariate was divided into two groups: non-depressive symptoms (< 5 points) and depressive symptoms (≥ 5 points). Additionally, the total wear time of the accelerometer was also collected and adjusted for this analysis.

### 2.4 Statistical analyses

In this study, IBM SPSS 23.0 (SPSS Inc., Chicago, IL, United States) was used to conduct statistical analyses. Descriptive analysis was used to present participants’ characteristics. The Chi-squared test was used to identify associations between covariates and cognitive function, and the level of significance was set at *P* < 0.05. Next, using baseline data, binary logistic regression analysis was performed to examine the association between independent variable, whether participants engaged in ≥ 3 h/day of LPA and, and dependent variables, MMSE scores (whether the overall and individual domains— orientation, attention and calculation, delayed recall, language, executive function, and visuoconstructional skills— of MMSE scores were at the maximum level) in two models: Model 1 was unadjusted; Model 2 was adjusted for age, sex, educational degree, wear time, MVPA time, and sedentary behavior time (variables significantly associated with cognitive function as determined by chi-square test analysis). Additionally, using baseline and follow-up data, we also used adjusted binary logistic regression to determine the longitudinal association between independent variable, whether participants engaged in ≥ 3 h/day of LPA at baseline, and dependent variables, MMSE scores (whether the overall and individual domains of MMSE scores maintained or increased) in the two models. Based on previous studies, our research uses 3 h as the cutoff point for LPA engagement time. To determine whether the score was maintained or increased, we subtracted the baseline MMSE scores from the follow-up MMSE scores for each domain of cognitive function. Model 1 was unadjusted; Model 2 was adjusted for age, sex, educational degree, wear time, MVPA time, and sedentary behavior time. Adjusted odds ratios (ORs) and 95% confidence intervals (CIs) were calculated for each variable. Further to that, an OR is the odds that an outcome will occur given a particular exposure, compared to the odds of the outcome occurring without that exposure. For instance, if the OR is 2.5 for participants who achieve 3 h/day of LPA, this means that those participants have 2.5 times the odds of maintaining or improving their cognitive function compared to participants who do not achieve 3 h/day of LPA.

## 3 Results

Table 1 presents characteristics of the participants and chi-square test results of covariates and cognitive functions. A total of 167 participants were included in this analysis. The means of participants were as follows: age, 76.11 ± 6.47 years; BMI, 24.40 ± 3.52 kg/m^2^; wear time per day, 14.48 ± 1.34 min; MVPA per day, 16.11 ± 25.16 min; LPA per day, 4.10 ± 1.37 h; Sedentary Behavior per day, 10.11 ± 1.26 h; baseline MMSE total score, 26.84 ± 3.49 points (orientation score, 9.22 ± 1.41 points; attention and calculation, 7.05 ± 1.43 points; delayed recall, 1.96 ± 0.99 points; language, 4.81 ± 0.51 points; executive function, 2.93 ± 0.28 points; visuoconstructional skills, 0.88 ± 0.33 points); and follow-up MMSE total score, 26.81 ± 4.06 points (orientation score, 9.13 ± 1.74 points; attention and calculation, 6.85 ± 1.59 points; delayed recall, 2.13 ± 0.91 points; language, 4.77 ± 0.58 points; executive function, 2.98 ± 0.15 points; visuoconstructional skills, 0.93 ± 0.26 points). Additionally, most participants were female (52.1%), were aged ≥ 75 years (51.5%), had an educational level above university (49.1%), had a normal BMI (52.1%), were living with others (88.0%), had four or more chronic diseases (10.2%), did not smoke (92.2%), did not drink alcohol (89.2%), did not engage in 150 min/week of MVPA (74.3%), engaged in ≥ 9 h/day of sedentary behavior (83.2%), had no depressive symptoms (82.6%), and engaged in ≥ 3 h/day of LPA (80.8%). Moreover, at baseline, 91.6% were not at risk of cognitive function impairment, compared to 88.6% at follow-up.

**TABLE 1:** Characteristics of the participants and Chi-square test results of covariates and cognitive functions (*n* = 167).

Categorical variables	Content	Total	*N*%	*P*-value for χ2 test* (pre-test)	*P*-value for χ2 test*(post-test)
Sex	Female	87	52.1%	0.038*	0.045*
	Male	80	47.9%		
Age	65–74	81	48.5%	0.007**	< 0.001**
	≥ 75	86	51.5%		
Educational level	No formal schooling	6	3.6%	< 0.001**	< 0.001**
	Elementary school	32	19.2%		
	Junior high school or above	129	77.3%		
BMI (kg/m^2^)	Underweight	2	1.2%	0.091	0.139
	Normal	83	49.7%		
	Overweight	50	29.9%		
	Obesity	32	19.2%		
Living alone	No	147	88.0%	0.149	0.836
	Yes	20	12.0%		
Having four or more chronic diseases	No	150	89.8%	0.596	0.452
	Yes	17	10.2%		
Smoking	No	154	92.2%	0.256	0.663
	Yes	13	7.8%		
Drinking alcohol	No	149	89.2%	0.174	0.410
	Yes	18	10.8%		
150 min MVPA per week	No	124	74.3%	0.105	0.119
	Yes	43	25.8%		
≥ 9 h/day of sedentary behavior	No	28	16.8%	0.795	0.154
	Yes	139	83.2%		
Depressive symptoms	No	138	82.6%	0.675	0.082
	Yes	29	17.4%		
≥ 3 h/day of LPA	No	32	19.2%	–	–
	Yes	135	80.8%		
Baseline cognitive function impairment	No	153	91.6%	–	–
	Yes	14	8.4%		
Follow-up cognitive function impairment	No	148	88.6%	–	–
	Yes	19	11.4%		
Continuous variables (independent variables)		Mean	SD	–	–
Age of baseline (years)		76.11	6.47	–	–
BMI (kg/m^2^)		24.40	3.52	–	–
Wear time per day (hours)		14.48	1.34	–	–
MVPA per day (minutes)		16.11	25.16	–	–
LPA per day (hours)		4.10	1.37	–	–
SB per day (hours)		10.11	1.26	–	–
**Continuous variables (dependent variables in baseline)**	**Mean**	**Normal overall cognitive function and max scores of domain-specific cognitive functions (participants engaging in ≥ 3 h/day of LPA)**		**Normal overall cognitive function and max scores of domain-specific cognitive functions (participants engaging in < 3 h/day of LPA)**	
Baseline MMSE total score	26.84	*n* = 127 (94.1%)		*n* = 26 (81.3%)	
Orientation	9.22	*n* = 92 (68.1%)		*n* = 17 (53.1%)	
Attention and calculation	7.05	*n* = 78 (57.8%)		*n* = 19 (59.4%)	
Delayed recall	1.96	*n* = 51 (37.8%)		*n* = 9 (28.1%)	
Language	4.81	*n* = 123 (91.1%)		*n* = 22 (68.8%)	
Executive function	2.93	*n* = 126 (93.3%)		*n* = 30 (93.8%)	
Visuoconstructional skills	0.88	*n* = 122 (90.4%)		*n* = 25 (78.1%)	
**Continuous variables (dependent variables in follow-up)**	**Mean**	**Maintenance or increase of overall and domain-specific cognitive functions (participants engaging in ≥ 3 h/day of LPA)**		**Maintenance or increase of overall and domain-specific cognitive functions (participants engaging in < 3 h/day of LPA)**	
Follow-up MMSE total score	26.81	*n* = 94 (69.6%)		*n* = 18 (56.3%)	
Orientation	9.13	*n* = 116 (85.9%)		*n* = 18 (56.3%)	
Attention and calculation	6.85	*n* = 100 (74.1%)		*n* = 23 (71.9%)	
Delayed recall	2.13	*n* = 108 (80.0%)		*n* = 26 (81.3%)	
Language	4.77	*n* = 123 (91.1%)		*n* = 30 (93.8%)	
Executive function	2.98	*n* = 131 (97.0%)		*n* = 32 (100%)	
Visuoconstructional skills	0.93	*n* = 132 (97.8%)		*n* = 29 (90.6%)	

*P-value for χ2 test < 0.05 represents a significant correlation;

**P-value for χ2 test < 0.01 represents a significant correlation; BMI, body mass index; LPA, light-intensity physical activity; MVPA, moderate-to-vigorous physical activity; SB, sedentary behavior.

In the Chi-squared test, a significance level of *P* < 0.05 and *P* < 0.01 were used; sex, age, and educational level were all significantly associated with cognitive function whether the other variable was pre-test overall cognitive function (impaired or not) or post-test overall cognitive function (impaired or not). We simultaneously adjusted for accelerometer wear time, MVPA time, and sedentary time per day (Ku et al., 2017) for analysis of PA. Therefore, we incorporated age, sex, educational level, wear time per day, MVPA time per day, and sedentary time per day as covariates for adjustment in the following analysis.

Table 2 presents the cross-sectional association between ≥ 3 h/day of LPA and cognitive function both overall cognitive function impairment and domain-specific cognitive function maximum levels using binary logistic regression models. In Model 1 (unadjusted), 3 h/day of LPA was significantly positively associated with overall cognitive function (OR: 3.66, 95% CI: 1.17–11.45; *P* = 0.026) and language (OR: 4.66; 95% CI: 1.80–12.10; *P* = 0.002), indicating that people who engage in 3 h/day of LPA are 3.66 times more likely to have better overall cognitive function compared to those who don’t and that less than a 5% chance that the observed association is due to random chance; in Model 2 (adjusted for covariates), LPA was not significantly associated with any domain of cognitive function.

**TABLE 2:** Binary logistic regression models examine the cross-sectional association between light-intensity physical activity (LPA) and cognitive functions (*n* = 167).

Scores reach maximum or not	3 h/day of LPA	Model 1		Model 2	
		**OR (95% CI)**	***P*-value**	**OR (95% CI)**	***P*-value**
Baseline overall cognitive function impairment	No Yes	1.00 3.66 (1.17, 11.45)	0.026*	1.00 1.36 (0.17, 10.60)	0.770
Orientation	No Yes	1.00 1.89 (0.86, 4.13)	0.112	1.00 0.94 (0.30, 2.95)	0.913
Attention and calculation	No Yes	1.00 0.94 (0.43, 1.05)	0.869	1.00 0.41 (0.13, 1.26)	0.119
Delayed recall	No Yes	1.00 1.55 (0.67, 3.61)	0.309	1.00 0.71 (0.20, 2.45)	0.583
Language	No Yes	1.00 4.66 (1.80, 12.10)	0.002*	1.00 3.08 (0.33, 29.18)	0.327
Executive function	No Yes	1.00 0.93 (0.19, 4.55)	0.932	1.00 1.92 (0.23, 16.30)	0.549
Visuoconstructional skills	No Yes	1.00 2.63 (0.95, 7.25)	0.062	1.00 0.94 (0.19, 4.66)	0.938

Model 1: unadjusted; model 2: adjusted for age, sex, educational degree, wear time, MVPA time, and sedentary behavior time; LPA, light-intensity physical activity; MVPA, moderate-to-vigorous physical activity; OR, odds ratio; CI, confidence interval.

*p < 0.05.

Table 3 presents the prospective association between 3 h/day of LPA and cognitive function using binary logistic regression models based on raw change score data obtained by subtracting baseline data from follow-up data. In Model 1 (unadjusted), LPA was significantly positively associated with the maintenance or increase of the orientation function (OR: 4.75; 95% CI: 2.03–11.11; *P* ≤ 0.001), indicating that people who engage in 3 h/day of LPA are 4.75 times more likely to maintain or improve their orientation function compared to those who don’t and that less than a 5% chance that the observed association is due to random chance; in Model 2 (adjusted for covariates), LPA was also significantly positively associated with the maintenance or increase of the orientation function (OR: 3.83; 95% CI: 1.01–14.46; *P* = 0.048), indicating that people who engage in 3 h/day of LPA are 3.83 times more likely to maintain or improve their orientation function compared to those who don’t and that less than a 5% chance that the observed association is due to random chance; LPA was significantly negatively associated with the maintenance or increase of the language function (OR: 0.88; 95% CI: 1.01–0.99; *P* = 0.049), indicating that people who engage in LPA are 0.88 times as likely to maintain or improve their language function compared to those who don’t and that less than a 5% chance that the observed association is due to random chance.

**TABLE 3:** Follow-up survey: binary logistic regression models examine the prospective association between light-intensity physical activity (LPA) and cognitive functions (*n* = 167).

Scores maintain or increase	3 h/day of LPA	Model 1		Model 2	
		**OR (95% CI)**	***P*-value**	**OR (95% CI)**	***P*-value**
Overall cognitive function maintain or increase	No Yes	1.00 1.78 (0.81, 3.93)	0.151	1.00 0.89 (0.30, 2.68)	0.835
Orientation maintain or increase	No Yes	1.00 4.75 (2.03, 11.11)	< 0.001**	1.00 3.83 (1.01, 14.46)	0.048*
Attention and calculation maintain or increase	No Yes	1.00 1.12 (0.47, 2.65)	0.800	1.00 0.71 (0.22, 2.32)	0.576
Delayed recall maintain or increase	No Yes	1.00 0.87 (0.35, 2.47)	0.923	1.00 0.65 (0.18, 2.33)	0.507
Language maintain or increase	No Yes	1.00 0.68 (0.15, 3.22)	0.630	1.00 0.88 (0.01, 0.99)	0.049*
Executive function maintain or increase	No Yes	1.00 0.00 (0.00, –)	0.998	1.00 0.00 (0.00, –)	0.998
Visuoconstructional skills maintain or increase	No Yes	1.00 4.55 (0.87, 23.70)	0.072	1.00 0.02 (0.00, 2.60)	0.116

Model 1: unadjusted; model 2: adjusted for age, sex, educational degree, wear time, MVPA time, and sedentary behavior time; LPA, light-intensity physical activity; MVPA, moderate-to-vigorous physical activity; OR, odds ratio; CI, confidence interval.

*p < 0.05.

**p < 0.001.

## 4 Discussion

To the best of our knowledge, this is the first study to examine the cross-sectional and longitudinal associations between accelerometer-measured LPA and both overall and the six domains of cognitive function measured using MMSE in a sample of older Taiwanese adults and adjusting for potential covariates. The main finding was that engaging in ≥ 3 h/day of LPA was beneficial to the maintenance or improvement of orientation in the long-term, regardless of the wear time of the accelerometer, time of sedentary behavior, and time of engagement in MVPA.

Our cross-sectional results revealed that LPA was not significantly associated with overall cognitive function, or all domains of cognitive function. This finding is consistent with previous research indicating an insignificant cross-sectional relationship between LPA and overall cognitive function measured using MMSE (Amagasa et al., 2020; Cavalcante et al., 2018; Fanning et al., 2017; Iso-Markku et al., 2018; Makizako et al., 2015; Marinac et al., 2015). It can be surmised that the effect of LPA on cognitive functions should be tracked over a long period of time; additionally, it can be explained that the risk factors for cognitive decline are multifactorial (World Health Organization, 2019), requiring in-depth research from multiple perspectives.

The main finding in this study corroborates previous findings that engaging in LPA is associated with reduced risks of cognitive function decline (Hsiao et al., 2022; Stubbs et al., 2017), and further indicates that engaging in ≥ 3 h/day of LPA could increase the chance of maintaining or improving the orientation function when compared with engaging in < 3 h/day of LPA. A possible explanation for the main finding of this study is that engaging in ≥ 3 h/day of LPA is associated with a lower white matter hyperintensity (WMH) volume (Spartano et al., 2019), which was very common findings on brain magnetic resonance imaging (MRI) or computed tomography (CT) scans in older adults and patients with stroke and dementia (Wardlaw et al., 2015); higher WMH volume exhibited significantly lower functional connectivity within the default-mode network (DMN) (Zhang et al., 2021), which mental orientation in space, time, and person is managed by Peer et al. (2015). Moreover, the result of a longitudinal study has indicated that physical activity was positively associated with the cortical connectivity within the DMN (Boraxbekk et al., 2016). Thus, lower white matter hyperintensity volume and higher connectivity within DMN may be factors influencing the orientation function of older adults that regularly engage in LPA. More specifically, the orientation function can be divided into two types in MMSE: time and spatial orientation. Time disorientation is related to global acute or chronic brain dysfunction, requiring bilateral lesions (Dumurgier et al., 2016). Spatial orientation can be categorized into several types — including landmark, egocentric, heading, and anterograde agnosia — related to the posterior parietal lobe of the brain, right hippocampus, and parahippocampal gyrus (Tseng and Fang, 2022). From the above, it is evident that to further investigate the physiological mechanisms linking LPA with specific domains of cognitive function, future research could focus on white matter and lateral ventricles. Also, future studies should further explore how LPA affects brain structures related to orientation.

The cross-sectional and longitudinal associations between engaging in ≥ 3 h/day of LPA and cognitive function are different. While engaging in ≥ 3 h of LPA was beneficial to language performance in the unadjusted model in the short-term, the longitudinal relationship became negatively significant between engagement in LPA and the maintenance or increase of the language score in the adjusted model. The reason may be first that the number of individuals with maximum scores of language domain in MMSE was already high in the cross-sectional findings (145/167 participants); the number of participants whose language performance remained unchanged or improved increased slightly to 155, causing the negative longitudinal relationship. Additionally, a longitudinal study demonstrated that orientation was the better domain to predict the overall MMSE score compared with other domains (Guerrero-Berroa et al., 2009), meaning that the language domain may be less discriminating. Moreover, previous research has also indicated the negative relationship between physical activity and language function, demonstrating that the comprehension speed of language of older adults became slower after the exercise training intervention, which was inferred to be related to the cost of language processing (Fernandes et al., 2024).

This study featured several strengths; first, objective instruments (triaxial accelerometers) were used to evaluate PA levels, enhancing the robustness and validity of the study results. Second, a comprehensive range of potential covariates in the analysis were included. Adjusting for these covariates underscored the robustness of the findings, supporting a significant positive longitudinal association between LPA and the orientation function. Third, our findings demonstrated that LPA specifically benefits orientation cognitive function, providing a basis for institutions to recommend it for maintaining or enhancing orientation cognitive function in older adults in a long term.

This study also had some limitations; first, due to time constraints and limited accelerometers, our study’s small sample size may cause wide confidence intervals, along with primarily urban participants with a low likelihood of cognitive impairment (from 8.4% at baseline to 11.4% at the follow-up) may affect the generalizability of the results to the overall population of older Taiwanese adults (Cassarino et al., 2016). Second, the sociodemographic, lifestyle behavior, and accelerometer-measured data were only collected at baseline, potentially overlooking changes over a year. Future research should control and analyze lifestyle changes (e.g., exercise participation). Third, using the same version of MMSE at baseline and follow-up may result in practice effects (Galasko et al., 1993). Also, the prognostic capability for cognitive impairment and dementia risk of MMSE is limited. Future research can use various other cognitive assessment tools, such as Trail Making Test, Digit Symbol Coding, Delayed Recall Tests, Rey Auditory Verbal Learning Test, and Repeatable Battery for the Assessment of Neuropsychological Status, to provide a more comprehensive understanding of participants’ cognitive status and avoid the ceiling effect. Lastly, our study calculated cognitive function changes by subtracting raw pre-test from raw post-test MMSE scores, potentially causing interindividual variability issues. And the high percentage of participants achieved maximum scores of cognitive functions, got maintained or increased scores, and engaged in ≥ 3 h/day of LPA, which may have influenced the results. While we examined the association between LPA and cognitive functions, we did not investigate differences between MVPA, LPA, and sedentary behavior, which should be explored further. Our research used binary logistic regression; future studies could use linear regression to better understand cognitive function variations.

## 5 Conclusion

This study demonstrated that engaging in ≥ 3 h/day of LPA was associated with the maintenance or improvement of the orientation function in older adults over a period of 1 year. Maintaining or increasing orientation cognitive function in a long term could be achieved by engaging in LPA according to this finding, particularly for older adults who are unable to participate in higher-intensity PA. As preliminary research, our study uniquely contributes to the literature by highlighting the specific benefit of LPA on orientation function, laying the groundwork for future research to explore these associations further and to investigate the impact on other domains of cognitive function.

## Data Availability

The raw data supporting the conclusions of this article will be made available by the authors, without undue reservation.
